# SEL120-34A is a novel CDK8 inhibitor active in AML cells with high levels of serine phosphorylation of STAT1 and STAT5 transactivation domains

**DOI:** 10.18632/oncotarget.16810

**Published:** 2017-04-04

**Authors:** Tomasz Rzymski, Michał Mikula, Eliza Żyłkiewicz, Agnieszka Dreas, Katarzyna Wiklik, Aniela Gołas, Katarzyna Wójcik, Magdalena Masiejczyk, Anna Wróbel, Izabela Dolata, Agata Kitlińska, Małgorzata Statkiewicz, Urszula Kuklinska, Krzysztof Goryca, Łukasz Sapała, Aleksandra Grochowska, Aleksandra Cabaj, Małgorzata Szajewska-Skuta, Ewelina Gabor-Worwa, Katarzyna Kucwaj, Arkadiusz Białas, Adam Radzimierski, Michał Combik, Jakub Woyciechowski, Maciej Mikulski, Renata Windak, Jerzy Ostrowski, Krzysztof Brzózka

**Affiliations:** ^1^ R&D Department, Selvita S.A., Kraków, Poland; ^2^ Department of Genetics, Maria Sklodowska-Curie Memorial Cancer Center, Warsaw, Poland; ^3^ Department of Gastroenterology, Hepatology and Clinical Oncology, Medical Center for Postgraduate Education, Warsaw, Poland; ^4^ Laboratory of Bioinformatics, Nencki Institute of Experimental Biology, Warsaw, Poland

**Keywords:** CDK8 mediator, kinase inhibitor, STAT5, AML, leukemia stem cells

## Abstract

Inhibition of oncogenic transcriptional programs is a promising therapeutic strategy. A substituted tricyclic benzimidazole, SEL120-34A, is a novel inhibitor of Cyclin-dependent kinase 8 (CDK8), which regulates transcription by associating with the Mediator complex. X-ray crystallography has shown SEL120-34A to be a type I inhibitor forming halogen bonds with the protein's hinge region and hydrophobic complementarities within its front pocket. SEL120-34A inhibits phosphorylation of STAT1 S727 and STAT5 S726 in cancer cells *in vitro*. Consistently, regulation of STATs- and NUP98-HOXA9- dependent transcription has been observed as a dominant mechanism of action *in vivo*. Treatment with the compound resulted in a differential efficacy on AML cells with elevated STAT5 S726 levels and stem cell characteristics. In contrast, resistant cells were negative for activated STAT5 and revealed lineage commitment. *In vivo* efficacy in xenotransplanted AML models correlated with significant repression of STAT5 S726. Favorable pharmacokinetics, confirmed safety and *in vivo* efficacy provide a rationale for the further clinical development of SEL120-34A as a personalized therapeutic approach in AML.

## INTRODUCTION

Two paralog kinases: CDK8 and CDK19, reversibly associate with the Mediator complex and form its key regulatory unit [1, 2]. Activities of CDK8 and CDK19 have been implicated in sustained proliferation and viability of cancer cell lines, probably by modulation of various gene expression programs. Currently, there is an interest in therapeutic targeting of these kinases in cancer, particularly in acute myeloid leukemia (AML) [3, 4]. Pharmacological inhibition of CDK8 and CDK19 by the steroidal alkaloid Cortistatin A (CA), deregulates expression of tumor suppressors and lineage-controlling genes in AML and results in suppressed growth of cancer cells [4]. Surprisingly, several reports have indicated that selective CDK8 inhibitors were inactive on colorectal cancer (CRC) cell lines with amplification of CDK8, which were previously characterized as being sensitive to CDK8 gene silencing [5, 6]. CDK8 activity is involved in WNT/β-catenin signaling [7], the serum response network [8], p53 pathway [9], p21 regulated transcription [10], Notch signaling [11] and a hypoxia response [12]. Several CDK8-selective probes displayed inhibitory activity for the WNT/β-catenin pathway *in vitro* in CRC cell lines [10, 13, 14]. In contrast, transcriptional profiling of AML cells treated with CA, revealed significant induction of super-enhancer (SE) associated genes [4]. Effects of CA on RNA polymerase II (RNAP II) transcription in the HCT-116 CRC line were rather modest and involved genes implicated in inflammation, growth, and metabolic regulation [6]. These effects only partially overlap with transcriptional profiling of CDK8 and CDK19 knockdown cells and the response to CA in AML cells [12, 15]. Such discrepancies were further discussed as a result of differences between kinase and scaffolding functions of CDK8 and CDK19 within the Mediator complexes [6].

Several substrates of CDK8 kinase have been identified [11, 16–18] and the majority of CDK8 inhibitors have been shown to consistently repress phosphorylation of the transactivation domains of STATs [4, 5, 14].

Here, we report characterization of a novel ATP-competitive and selective CDK8 inhibitor SEL120-34A, with an unusual binding mode compared to other CDK8 inhibitors [19]. In keeping with previous studies, SEL120-34A inhibited phosphorylation of STAT1 at serine 727 (S727) and STAT5 at serine 726 (S726) in AML cells. Efficacy studies of SEL120-34A and other structurally non-related CDK8 inhibitors in AML cells indicated differential activity on cells positive for phosphorylated STAT1 S727 and STAT5 S726. Transcriptional profiling of SEL120-34A effects revealed selective activity on genes regulated by STATs and NUP98-HOXA9 signaling. High bioavailability after oral administration and metabolic stability enabled *in vivo* efficacy studies, which indicated AML tumor growth inhibition at safe doses. Taken together, SEL120-34A is a first in class CDK8 inhibitor which has advanced into preclinical development and may be a convenient tool for further biological studies.

## RESULTS

### SEL120-34A is a novel selective CDK8 inhibitor

**Figure 1:**
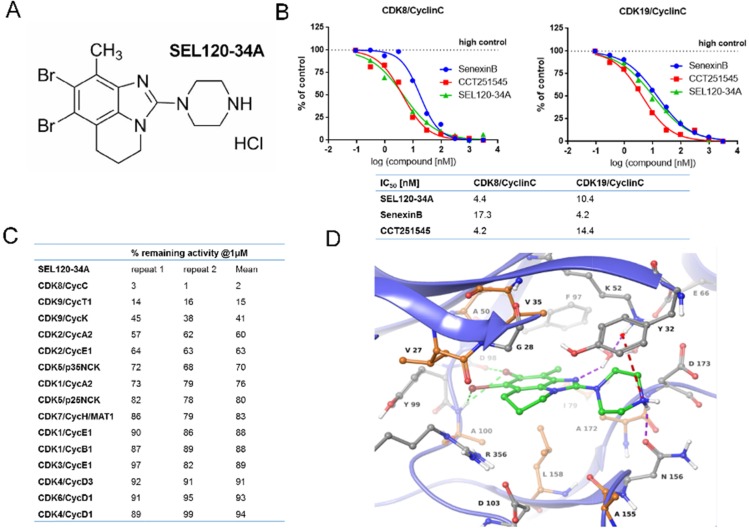
Structure and activity of SEL120-34A **(A)** Chemical structure of SEL120-34A. **(B)** The IC_50_ of SEL120-34A, Senexin B and CCT241545 determined by constructing a dose-response curve and examining inhibition of CDK8/CycC and CDK19/CycC activities at Km ATP concentrations. **(C)** % remaining activities measured for members of the CDK family in the presence of 1 μM SEL120-34A at Km ATP concentrations. **(D)** Active site of the crystal structure of human CDK8/CycC complexed with SEL120-34A. Protein residues and SEL120-34A are shown as Ball-and-Sticks. Protein carbon atoms are colored orange (aliphatic hydrophobic residues) or gray (other residues), while ligand carbon atoms are colored green. The following interactions are shown: H bond as purple dashed line, halogen bonding as green dashed line and cation-*π* system interaction as red dashed line.

Structure-based drug design led to the synthesis of a substituted tricyclic benzimidazole SEL120-34A as a novel CDK8 inhibitor (Figure 1A). The detailed synthesis pathway is available in the supplementary methods. We determined that SEL120-34A inhibited kinase activities of CDK8/CycC and CDK19/CycC complexes with an IC_50_ of 4.4 nM and 10.4 nM, respectively (Figure 1B). The dissociation constant (Kd) for the CDK8 protein was estimated at 3 nM (Supplementary Figure 1). These values were comparable with two other, structurally unrelated CDK8 inhibitors, namely Senexin B (SNX2-1-165 from patent WO-2014134169) [20], and CCT251545 [21] (Figure 1B and Supplementary Figure 1). By contrast, SEL120-34A did not significantly inhibit other members of the CDK family in a single point inhibition assay, namely CDK1, 2, 4, 6, 5, 7 *in vitro* (Figure 1C), with the exception of CDK9, however a calculated IC_50_ 1070 nM, indicated an over 200 fold selectivity against this kinase (Supplementary Figure 2).

### Binding mode of SEL120-34A

To understand the binding mode of SEL120-34A to CDK8, we resolved a 2.8-Å crystal structure of the CDK8/CycC/SEL120-034A complex. We observed inhibitor binding to the kinase in DMG-in conformation, similar to the previously reported structures of CDK8/CycC alone, complexed with CA or in complex with a small molecule inhibitor of WNT signaling [4, 14, 21, 22]. SEL120-34A interacts with the ATP binding site of CDK8 in a type I inhibitor manner by forming several types of interactions with the protein (Figure 1D). Two bromine atoms form halogen bonds with the carbonyl group of Asp 98 and the backbone's NH of Ala 100. Additionally, one bromine atom interacts with the π-system of Tyr 99. The piperazine moiety is stacked between side chains of Tyr 32 and Asn 156. Furthermore, its amine moiety forms the following interactions: (i) an ionic interaction with a carboxyl group of Asp 173, (ii) a hydrogen bond with a side chain carbonyl group of Asn 156 and (iii) a cation-π system interaction with Tyr 32. The nitrogen atom of the benzimidazole core forms a water mediated hydrogen bond with Lys 52. The methyl group of SEL120-34A points toward the gatekeeper residue (Phe 97) and Ile 79. The tricyclic benzimidazole core forms hydrophobic contacts with “the floor” (Ala 100, Leu 158, Ala 172) and “the ceiling” (Val 27, Gly 28, Val 35, Ala 50) of the ATP binding pocket. Additionally, a partially aliphatic ring of the benzimidazole core forms contacts with Arg 356 that constitutes the side wall of the pocket. The latter is a key for selectivity, as Arg 356 is a part of a C-terminal extension unique for CDK8. The observed protein-ligand interaction pattern obeys rules for long residence time, namely H-bond formation with the hinge region (here exhibited by halogen bonding) and hydrophobic complementarities within the front pocket [22].

### SEL120-34A inhibits phosphorylation of STAT1 S727 and STAT5 S726

**Figure 2:**
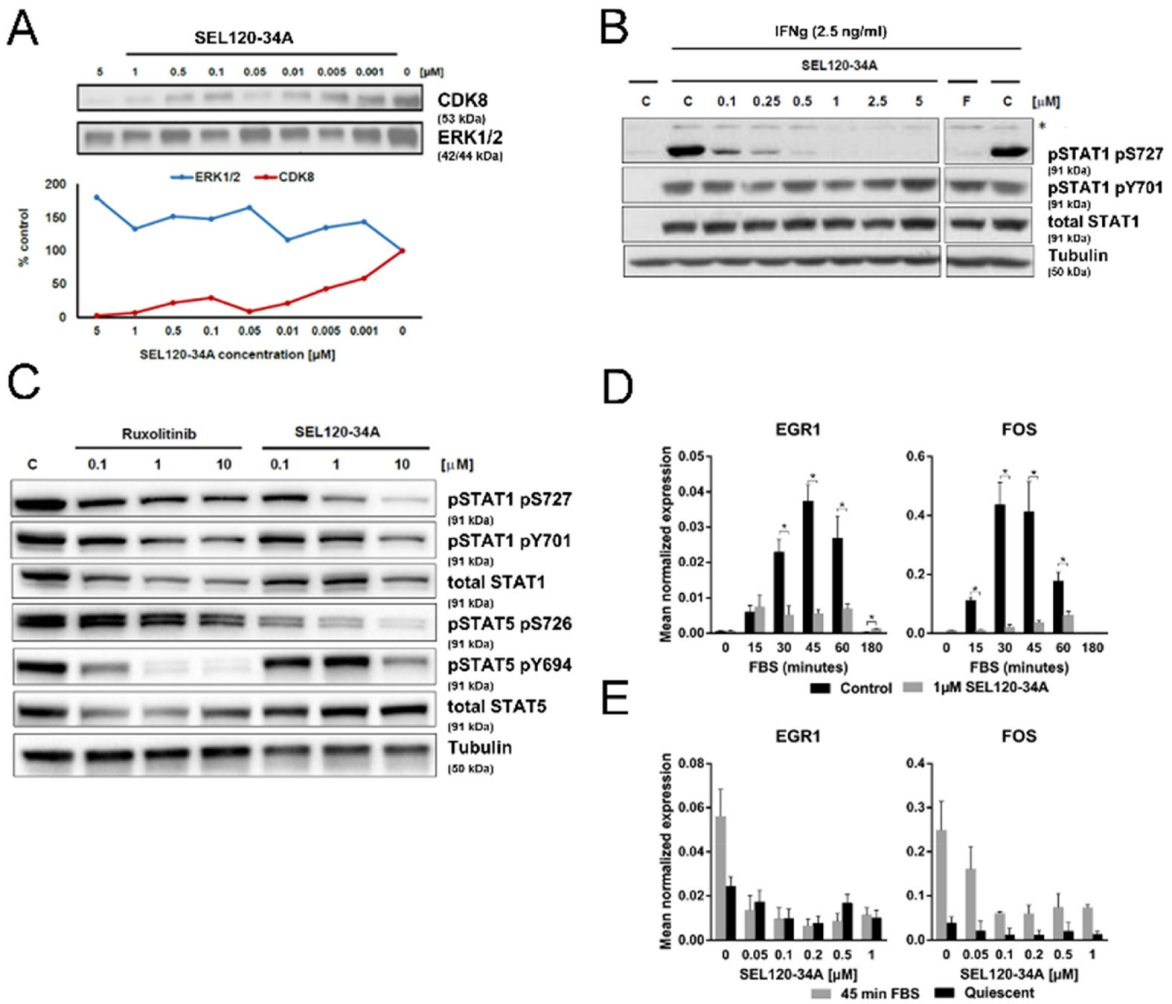
SEL120-34A inhibits phosphorylation of STAT1 S727 and STAT5 S726 and mitogen induced expression of immediate early response genes **(A)** SEL120-34A competitively inhibits CDK8 binding to ATP -desthiobiothin probes (ActivX) in KG-1 AML cell lysate; ERK1/2 was used as a control. CDK8 and ERK1/2 proteins in precipitates were revealed by WB. **(B)** SEL120-34A inhibits IFN-dependent phosphorylation of STAT1 S727. HCT-116 cells stimulated with IFNg for 4 h were treated with increasing doses of SEL120-34A, Flavopiridol (F) or vehicle DMSO **(C)** to determine changes in the phosphorylation of STAT1 S727 and Y701, as measured by WB. (*) unspecific band. **(C)** SEL120-34A inhibits serine phosphorylation of STAT1 S727 and STAT5 S726, whereas Ruxolitinib inhibits tyrosine phosphorylation of STAT1 Y701 and STAT5 Y694 in JAK2 V617F cells. SET-2 cells were treated with Ruxolitinib, SEL120-34A or vehicle DMSO **(C)** for 24 hours and protein levels of pS727, pY701 STAT1 and pS726, pY694 STAT5 were measured by WB. **(D)** SEL120-34A inhibits serum- induced mRNA expression of immediate early response genes in a dose- dependent manner.HCT-116 cells were synchronized in 0.5% FBS medium for 24 h, cells were pretreated for 120 minutes with 1 μM SEL120-34A followed by activation with 10% FBS and subsequent RNA extraction, DNAse I treatment and qRT-PCR reaction. The results were normalized for RPLP0 mRNA (means ±S.D., n=4). Statistical analysis of differences between mRNA levels for control and SEL120-34A at the indicated time point was performed using t-tests. A p value of ≤ 0.05 (*) was considered significant. **(E)** HCT-116 cells were prepared as in panel *D* and then challenged for 120 min with increasing doses of SEL120-34A, followed by the treatment with 10% FBS 45 min. RNA was extracted, DNAse I digested and subjected to the qRT-PCR reaction. The results were normalized for RPLP0 mRNA (means ±S.D., n=4).

We evaluated the affinity of SEL120-34A to CDK8 in KG-1 AML cell lysates using desthobiotin-ATP probes. SEL120-34A could effectively bind to CDK8 in a dose-dependent manner (at 1- 100 nM concentration range) as shown by competition with ATP- analogue probes (Figure 2A). CDK8 kinase specifically targets S727 phosphorylation at the transactivation domain of STAT1 upon interferon γ (IFNg) treatment [17]. This modification is not required for the formation of STAT dimers, however it was found to trigger full transcriptional activity of STAT proteins [23]. To test if SEL120-34A alters S727 phosphorylation, we first treated HCT-116 CRC cells with increasing doses of IFNg or interferon α/β (INFa). As expected, IFN stimulation enhanced phosphorylation of STAT1 S727 and tyrosine 701 (Y701) (Supplementary Figure 3A and 3B). We next challenged HCT-116 cells with IFNa or IFNg for 4 hours in the presence of increasing concentrations of SEL120-34A, which resulted in a dose-depended inhibition of S727, that was more pronounced after IFNg treatment (Figure 2B and Supplementary Figure 3C). A similar reduction in STAT1 S727 phosphorylation was observed after treatment with the pan-CDK inhibitor Flavopiridol. The C-terminal repeat domain (CTD) of RNAP II phosphorylation at S2 and S5 [24, 25] was not affected by up to 2.5 μM of the compound in cells (Supplementary Figure 4), indicating selectivity over CDK9, whereas Flavopiridol was active towards RNAP II.

The relationship with Janus kinase (JAK)-dependent activation of STATs was further studied in SET-2 cells which carry the JAK2 V617F mutation [26]. JAKs activate STATs by phosphorylating their conserved C-terminal tyrosine residues. Treatment with Ruxolitinib, a specific inhibitor of JAK2, resulted in the inhibition of STAT5 Y694 phosphorylation, whereas S726 was unaffected. In contrast, SEL120-34A exhibited activity towards S726, but not Y694 (Figure 2C).

### SEL120-34A inhibits mitogen- induced expression of immediate early response (IER) genes

Earlier studies on the CRC cell line HCT-116 provided evidence on CDK8 involvement in IER gene expression under mitogen stimulation [8]. We challenged quiescent HCT-116 cells for 2 hours with 1 μM SEL120-34A, followed by a time-course stimulation with FBS. SEL120-34A substantially repressed inducible expression of well-established IER genes, EGR1 and FOS (Figure 2D) [27]. Further assessment of IER genes showed a dose-dependent inhibition of EGR1 and FOS mRNA expression at 45 min of FBS stimulation (Figure 2E). In agreement with previous studies [8], these results indicate that pharmacological inhibition of CDK8 alters inducible IER gene expression.

### SEL120-34A inhibits IFN- responsive genes expression *in vitro*

**Figure 3:**
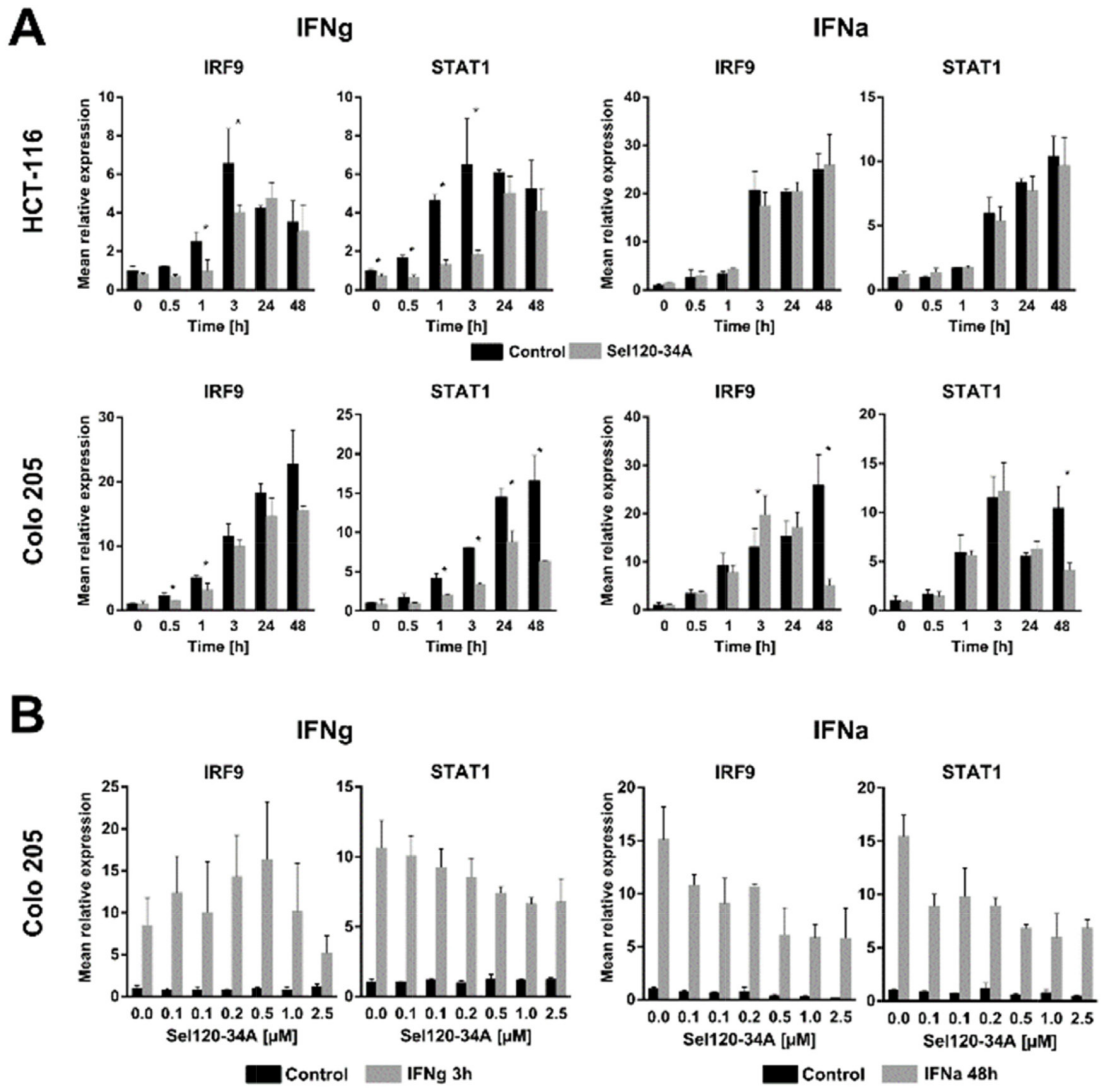
SEL120-34A inhibits interferon- induced genes expression in a dose dependent manner **(A)** HCT-116 and Colo-205 cells were made quiescent overnight by decreasing FBS in medium to 0.5% and then preincubated with 0.5 μM SEL120-34A for 1 h followed by either IFNg (5 ng/mL) or IFNa (250 units/mL) stimulation in the presence of SEL120-34A. At the indicated time point, cells were harvested, RNA extracted and transcripts levels analyzed by the qRT-PCR reaction. The results were normalized for RPLP0 mRNA (means ±S.D., n=3). **(B)** Colo-205 cells were synchronized as in panel *A* and then challenged with increasing doses of SEL120-34A for 1 h followed by either 3 h IFNg (5 ng/μl) or 48 h IFNa (250 units/ml) stimulation. At this time RNA was extracted, DNAse I digested and subjected to the qRT-PCR reaction. The results were normalized for RPLP0 mRNA (means ±S.D., n=3). Differences between mRNA levels for control and SEL120-34A at the indicated time points were assessed by t-tests. A p value of ≤ 0.05 (*) was considered significant.

In order to determine whether SEL120-34A inhibits expression of well-established IFN-responsive genes, we measured expression of STAT1 and IRF9, since these were shown to be induced in CRC cell lines by both types of IFNs [28]. HCT-116 and Colo-205 were pretreated with SEL120-34A for 1 hour and then subjected to a IFNg or IFNa time-course. SEL120-34A significantly decreased IRF9 and STAT1 mRNA expression in at least one early (0-3 h) and late (24 h-48 h) time point in IFNg treated cells, whereas for IFNa, such a decrease was only clearly pronounced at the 48 h time point (Figure 3A). Next, we focused on 3 h and 48 h time points for IFNg and for IFNa, respectively. Treatment with increasing amounts of the drug demonstrated that the STAT1 transcript is modulated in a dose-dependent manner in both IFNs stimulations, while the same effect on IRF9 transcript became pronounced after a 48 h IFNa treatment (Figure 3B).

Collectively, these results suggest that SEL120-34A inhibits IFN-dependent gene expression, however the effects on these genes are modest and transient, indicating that CDK8 is not a major driver of STAT-dependent transcription in IFN-stimulated cancer cells *in vitro*.

### Transcriptome profiling reveals specific alterations in interferon- responsive genes after SEL120-34A treatment *in vivo*

**Figure 4:**
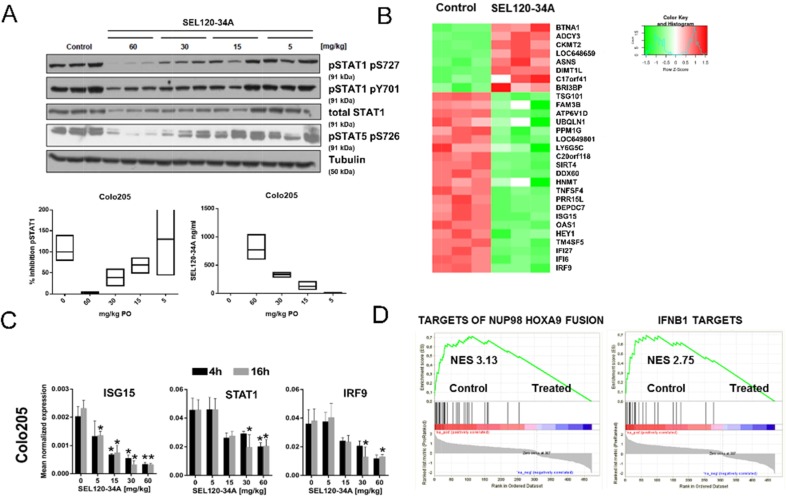
Xenografts transcriptome profiling reveals alteration in IFN- responsive genes upon SEL120-34A treatment *in vivo* **(A)** Animals xenografted with Colo-205 tumors were dosed orally with 5, 15, 30 and 60 mg/kg BID for a total of 3 days and tumor samples were dissected 16 hours after the last compound dose. Water was used as a vehicle. Levels of phosphorylated STAT1 S727, Y701 and STAT5 S726 were measured by WB. Floating bars plots indicate relative inhibition of STAT1 S727, measured by densitometry of WB signals (left) and corresponding concentrations of SEL120-34A detected in plasma of treated animals, measured by LC-MS (right). Mean, min and max values are presented. **(B)** Heat map showing changes in expression levels of the top 30 differentially expressed genes (based on adj. p-values, Z-scores are shown) in Colo-205 tumors, after treatment with 30 mg/kg of SEL120-34A at 4h time point **(C)** Validation of microarray data by qRT-PCR for selected IFN-regulated genes. Animals were administered with 5, 15, 30 and 60 mg/kg BID for 3 days and Colo-205 tumor samples were collected at 4 and 16 hours after the last compound dose. Total RNA was extracted and subjected to qRT-PCR measurements. mRNA expressions of measured transcripts were normalized to RPLP0 expression (n=3;±SD). Differences between mean cDNA levels for control and a given SEL120-34A dose at the indicated time point were assessed by t-tests. A p-value of ≤ 0.05 (*) was considered significant. **(D)** GSEA running plots for expression changes in Colo-205 xenografts after SEL120-34A treatment. Normalized Enrichment Scores (NES) for the selected sets from the MSigDB C2 collection are shown – (Takeda Targets of NUP98-HOXA9 Fusion 3d Upand Hecker IFNB1 Targets).

We studied STAT5 S726 and STAT1 S727 in Colo-205 CRC xenograft tissues in mice, treated orally with increasing doses of SEL120-34A (Figure 4A). Nearly complete inhibitions of STAT1 S727 and STAT5 S726 were observed at 60mg/kg, which resulted in a mean plasma concentration of the compound over 700 ng/ml at the 16 hour time point (Figure 4A).

It was plausible to expect that favorable pharmacodynamics of SEL120-34A could be sufficient to affect expression of CDK8 and CDK19- dependent genes *in vivo*. Whole transcriptome measurements were performed on Colo-205 xenograft tissues challenged with 30 mg/kg SEL120-34A at 4 and 16 hours after the last dose. This analysis revealed 678 differentiating (*p_adj_*≤ 0.05) probe sets in SEL120-34A treated Colo-205 (Supplementary Table 3). Manual inspection of the top differentially expressed genes (Figure 4B) indicated that the majority of genes repressed by the treatment were previously described as being STAT-regulated, including IRF9, IFI6, IFI27, TM4SF5, HEY1, OAS1 and ISG15; among many others [29]. The top 10% of differentially expressed probe sets (highest corrected p-value = 0.008) were then used for calculating their attribution to GO terms, which uncovered significant (*p_adj_*≤ 0.05) over-representation of genes belonging to IFN signaling pathways (Supplementary Table 4). To confirm the expression changes from microarrays, we demonstrated inhibition of STAT1, ISG15 and IRF9 in xenografts challenged with increasing doses of SEL120-34A by RT-qPCR. (Figure 4C). Reduced mRNA levels of STAT1 could also explain lower levels of STAT1 protein observed in treated tumors.

We next used GSEA [30] to investigate previously identified CDK8-dependent alterations, including SE-associated signatures [4] and curated gene sets (C2 v.5.1) from MSigDB. The highest enrichment scores were observed for IFN - target genes (NES 2.7) (Figure 4D) and for genes regulated by NUP98-HOXA9 – a frequent target of chromosomal rearrangements in leukemia (NES 3.1) [31–34]. Surprisingly, GSEA did not revealed significant enrichment of many signatures which were identified previously as being CDK8-dependent, including WNT/β-Catenin and SE-gene sets, identified previously in AML [4, 7] (Supplementary Table 5). Overall, these studies confirmed SEL120-34A as a specific and robust inhibitor of STATs-dependent gene expression *in vivo*.

**Figure 5:**
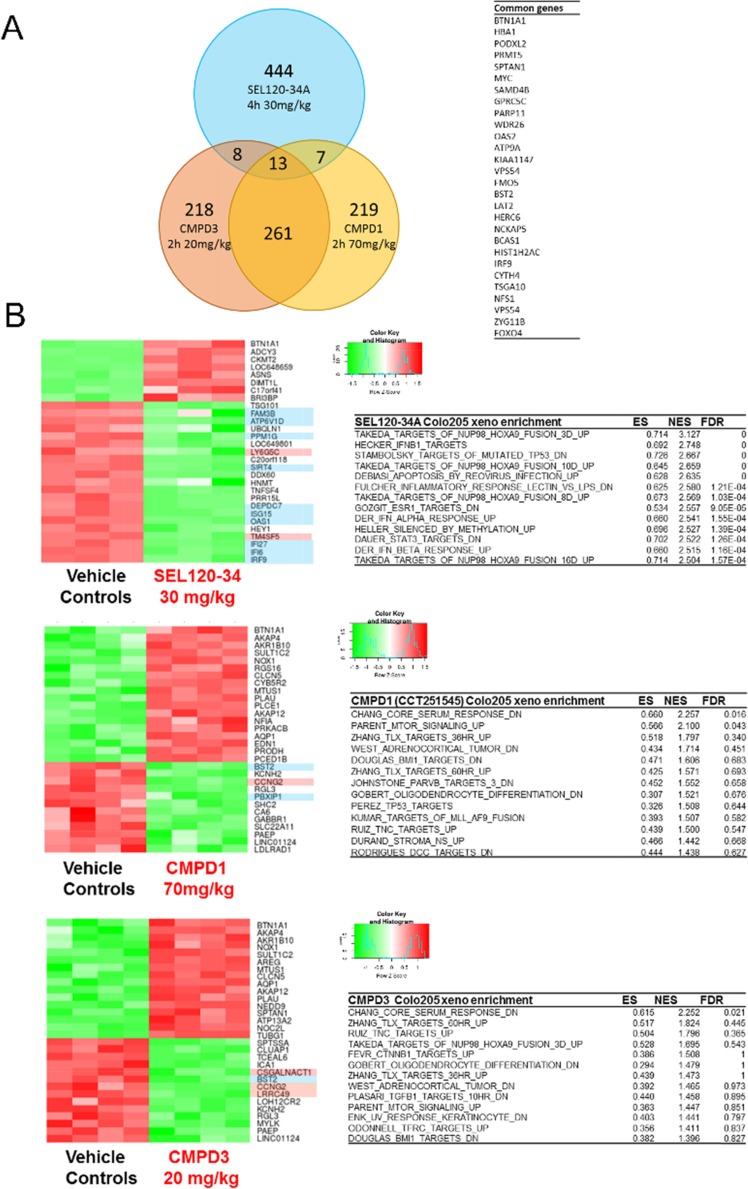
Transcriptional profiling of Colo-205 xenografted tumors revealed differences between SEL120-34A and two other CDK8 inhibitors - CCT251545 (CMPD1) and CMPD3 **(A)** Venn diagram showing overlap in significantly regulated genes by SEL120-34A 30mg/kg, CCT251545 (CMPD1) 70mg/kg and CMPD3 20mg/kg (Clarke et al.) based on adj. p-values. A list of common genes between SEL120-34A and both CMPD1 and CMPD3 is shown in tabular form. **(B)** Heatmaps presenting Z-scores for the top list of genes regulated in Colo-205 tumors by the treatment with SEL120-34A, CCT251545 (CMPD1) and CMPD3 (Clarke et al.). Gene names with a blue background indicate presence of STAT binding sites and red indicate HOXA binding sites in the promoters as analyzed by SABiosciences' database DECODE. Tables present GSEA analysis (MSigDB C2 collection) for expression changes in Colo-205 xenografts after treatments with indicated compounds, Enrichment Scores (ES), Normalized Enrichment Scores (NES) and False Discovery Rates (FDR) are shown.

Recently Clarke et al. also published microarray gene expression profiling of Colo-205 tumors treated with two distinct CDK8 inhibitors CCT251545 (described as CMPD1) and CMPD3 [35]. Despite comparable affinity to CDK8 *in vitro* and pharmacodynamics *in vivo*, only a ~5% overlap was observed between affected genes (Figure 5A). Notably, the top upregulated gene - BTN1A1, was the same for all three compounds (Figure 5A, 5B). In contrast to CMPD1 and CMPD3, the majority of genes downregulated by SEL120-34A contained either STAT1 or HOXA binding elements (Figure 5B). Moreover GSEA had not identified significant enrichment in STATs and IFN – dependent genes among these regulated by CCT251545 (CMPD1) and CMPD3 *in vivo* (Figure 5B). Instead genes related to the activity of TLX, BMI, TNC and CTNBB1 were affected, which were absent in SEL120-34A treated tumors.

### Efficacy of pharmacological inhibition of CDK8 on cancer cells

**Figure 6:**
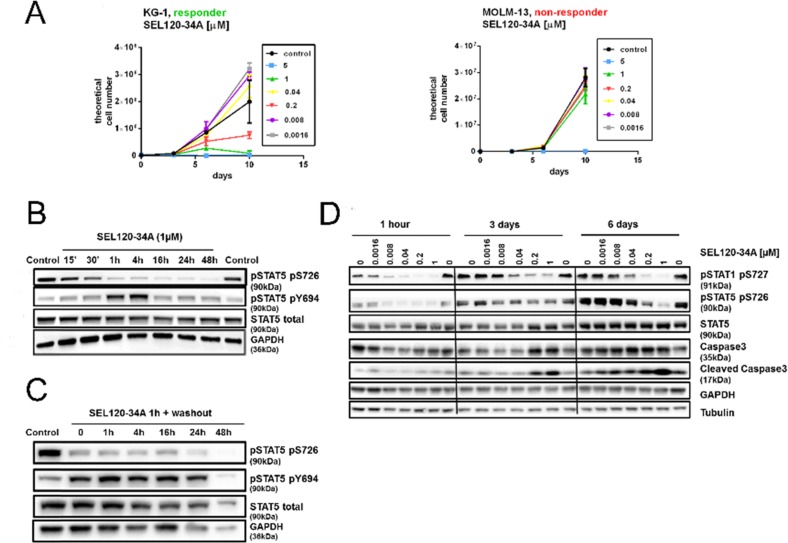
Efficacy of SEL120-34A in responder AML cells lines coincides with the inhibition of STAT5 S726 **(A)** Growth curves of responder KG-1 cell line and non-responder MOLM-13 cell line treated with increasing concentrations of SEL120-34A **(B)** Time-dependent effects of 1 μM SEL120-34A in KG-1 cells. Levels of STAT5 and phosphorylated STAT5 S726 and Y694 were measured by WB. **(C)** STAT5 S726 inhibition after the wash-out, following 1 h treatment with 1 μM SEL120-34A. **(D)** Effects of prolonged SEL120-34A exposures in KG-1 cells. KG-1 were treated with SEL120-34A for 1 h, 3 days and 6 days and levels of total STAT5, STAT5 S726, STAT1 S727, Caspase 3 and Cleaved Caspase 3 were measured by WB.

**Figure 7:**
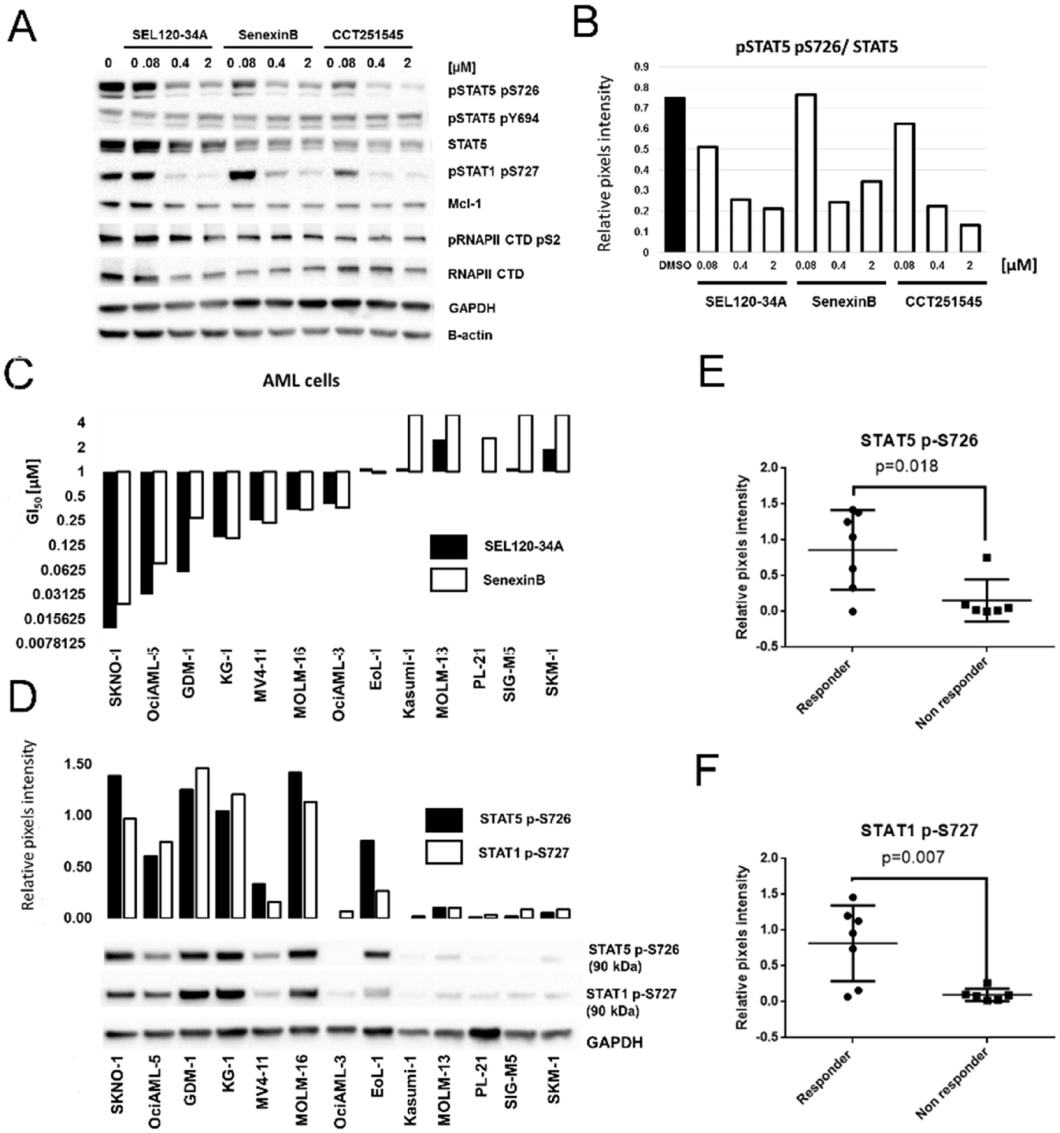
Treatment with CDK8 inhibitors results in differential activity on AML cell lines positive for phosphorylated STAT5 S726 and STAT1 S727 **(A)** KG-1 cells were treated for 24 h with SEL120-34A, Senexin B and CCT251545 and levels of total STAT5, phosphorylated STAT5 S726, Y694, phosphorylated STAT1 S727, MCL-1, total RNAPII CTD and phosphorylated RNAPII CTD S2 were measured by WB. **(B)** Densitometry showing relative repression of phosphorylated STAT5 S726 (normalized to total STAT5) by CDK8 inhibitors from panel A. **(C)** Effects of SEL120-34A and Senexin B on the growth of indicated AML cell lines, measured by extended viability tests. Mean GI_50_ (day 10) is shown and plotted on the graph (n=3). **(D)** Levels of phosphorylated STAT5 S726 and STAT1 S726 in AML cells from the panel *C* were measured by WB. Densitometry for STAT5 S726 and STAT1 S726 signals is shown and plotted on the graph. Cell lines from the panel *D* were divided into two classes based on the sensitivity to CDK8 inhibitors treatment (responders GI_50_<1 μM and non-responder GI_50_>1 μM), mean levels of STAT5 S726 **(E)** or STAT1 S727 **(F)** in selected cell lines measured by densitometry were presented on scatter plots. Differences between responder and non-responder were assessed by t-tests (p value is shown).

Our studies and those of other groups indicated moderate or low activity of known CDK8 inhibitors on CRC cell lines *in vitro*, therefore we have focused on leukemia cells which were found to be sensitive to CA [4]. Previous experiments indicated that SEL120-34A could repress phosphorylation of STAT5 which has been implicated in the maintenance of leukemia stem cells [36]. First, we determined that STAT5 S726 positive KG-1 AML cells were sensitive to prolonged treatment with SEL120-34A (Figure 6A, also compare with the Figure 7D), whereas S726 negative MOLM13 AML cells (Supplementary Figure 5A, compare with the Figure 7D) were resistant. Maximal inhibition of STAT5 S726 in KG-1 cells was achieved rapidly, after 1 hour of treatment (Figure 6B). Extended inhibition of STAT5 S726 by SEL120-34A was observed in the washout experiment, indicating long cellular residence time (Figure 6C). Continuous treatment with the compound in KG-1 cells showed sustained inhibition of STAT5 S726 and STAT1 S727 until day 6 and the hallmarks of apoptosis, such as cleaved Caspase-3, particularly at later time points (Figure 6D).

To determine whether pharmacological inhibition of CDK8 regulates viability of leukemia cells, we used SEL120-34A and two other CDK8 inhibitors, namely Senexin B [20], and CCT251545 [21]. All these compounds effectively bind to CDK8/CycC (Supplementary Figure 1A) and could potently inhibit phosphorylation of STAT5 S726 and STAT1 S727 in KG-1 AML cell line (Figure 7A, 7B). At the same time RNAPII CTD S2 and MCL1, which are strongly dependent on the activity of CDK9 in leukemia cells, were not affected [37]. Overall, the lack of structural similarities, comparable affinity to CDK8 and selectivity of these probes enabled studies on CDK8-related processes in leukemia cells.

Broad profiling of SEL120-34A and Senexin B in a panel of AML cell lines revealed several sensitive (GI_50_<1 μM)- responder cell lines (SKNO-1, KG-1, HEL-60, MOLM-16, MV-4-11, OciAML-2, MOLM-6 and OciAML-3), consistent with the effective inhibition range of STAT1 S727 and STAT5 S726 (Figure 7C). Other cells were considered as non-responders when growth inhibition GI_50_ exceeded 1 μM. A differential sensitivity pattern was further corroborated by treatment with CCT251545 (Supplementary Figure 6). Surprisingly, all three CDK8 inhibitors were inactive on the MOLM-14 cell line, which was previously identified as sensitive to CA treatment by a mechanism involving deregulation of genes under the control of SE [4]. We excluded misidentification of this and all other cell lines by the short tandem repeat analysis.

### Sensitivity of AML cell lines to the treatment with CDK8 inhibitors correlates with the presence of STAT5 S726 and STAT1 S727

Previous studies have demonstrated that cells sensitive to CA treatment responded by deregulation of SE-dependent genes, however discriminating markers which could predict sensitivity to CDK8 inhibitors have not been identified [4]. Our studies confirmed differential activity of selective CDK8 inhibitors on leukemia cells *in vitro*. Thus, we divided AML cell lines into two classes based on their sensitivity to CDK8 inhibitor treatment (responders and non-responders) and then analyzed levels of phosphorylated STAT1 and STAT5 in these cells (Figure 7D). Clearly, a group of responder AML cell lines was characterized by significantly higher levels of STAT5 S726 (Figure 7E) and STAT1 S727 (Figure 7F), whereas cell lines resistant to CDK8 inhibitors were largely negative for these phosphorylation marks.

**Figure 8:**
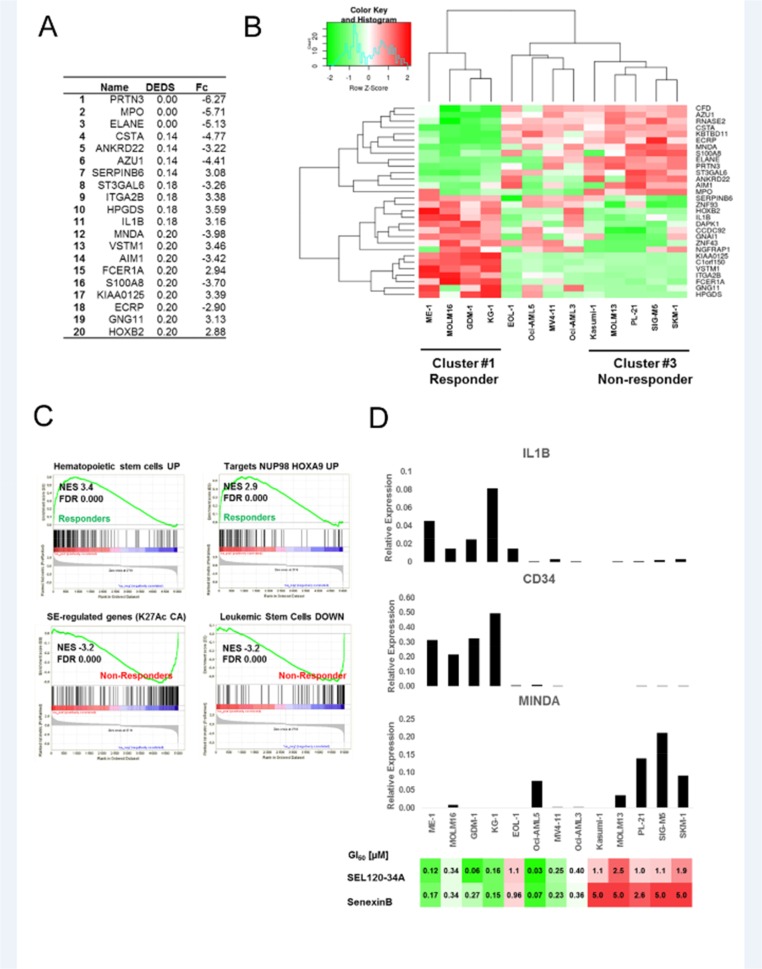
Identifying discriminating genes that can be used for predicting response to CDK8 inhibitors **(A)** Alist of the top 20 genes discriminating between CDK8 inhibitors responder and non-responder AML cells, ranked by DEDS scores, fold changes are shown (Fc). **(B)** Heat map showing results of clustering (Z-scores) for top discriminating genes between responder and non-responder AML cells. **(C)** GSEA for two classes: responder and non-responder cells. Running plots for sets (MSigDB C2 collection) with the most significant enrichment scores (NES) in both responder and non-responder classes are shown. **(D)** CD34, IL1B and MNDA mRNA expression levels were analyzed in AML cell lines from the panel *B* by qRT-PCR. Relative expression levels normalized to GAPDH are shown (representative biological repeat n=2, mean values from technical repeats n=3). Heat map presenting GI_50_ values for tested AML cells lines treated with SEL120-34A and Senexin B in extended viability assays is presented below for a comparison with gene expression data.

Our next objective was to identify sets of discriminating genes that could be used for characterizing and predicting response to CDK8 inhibitors. We identified differentially expressed genes between responder and non-responder AML cell lines (Figure 8A) using expression data from the Cancer Cell Line Encyclopedia (CCLE) [38] and ranked them using p-values from multiple statistical models for evidence of differential expression (DEDS) [39]. Clustering of genes with the highest DEDS scores, classified analyzed AML cell lines into 3 groups: non-responder cells with high expression of genes indicating myeloid commitment such as MPO, MNDA and ELANE and two distinct responder classes (Figure 8B). The first responder class was characterized by the high expression of marker genes for hematopoietic progenitor and stem cells, such as HOXB2 and ITGA2B (CD41) and IL1B [40, 41]. RT-qPCR analysis of discriminating genes confirmed elevated expression of MNDA in non-responder cells with low STAT5 S726 levels, whereas IL1B was highly elevated in the first cluster of responder cells with high STAT5 S726 levels (Figure 8D). CD34, which is well established marker of hematopoietic and leukemia stem cells [42–44], was detectable only in the first responder cluster.

GSEA using SE-associated signatures [4] and curated gene sets (C2 v.5.1) from MSigDB confirmed high enrichment of genes associated with hematopoietic stem cells (NES 3.4) and NUP98-HOXA9-target genes (NES 2.9) in responder cells, relative to non-responders AML cells (Figure 8C and Supplementary Table 6). In contrast, a high enrichment of genes downregulated in leukemic stem cells was observed in non-responder cells (NES -3.2). Interestingly, non-responder cells were also characterized by a high enrichment of genes classified by Pelish et al. as genes with high levels of histone acetylation and modulated by CA in MOLM-14 cells (Figure 8C and Supplementary Table 7).

### Efficacy of SEL120-34A in AML xenograft models

**Figure 9:**
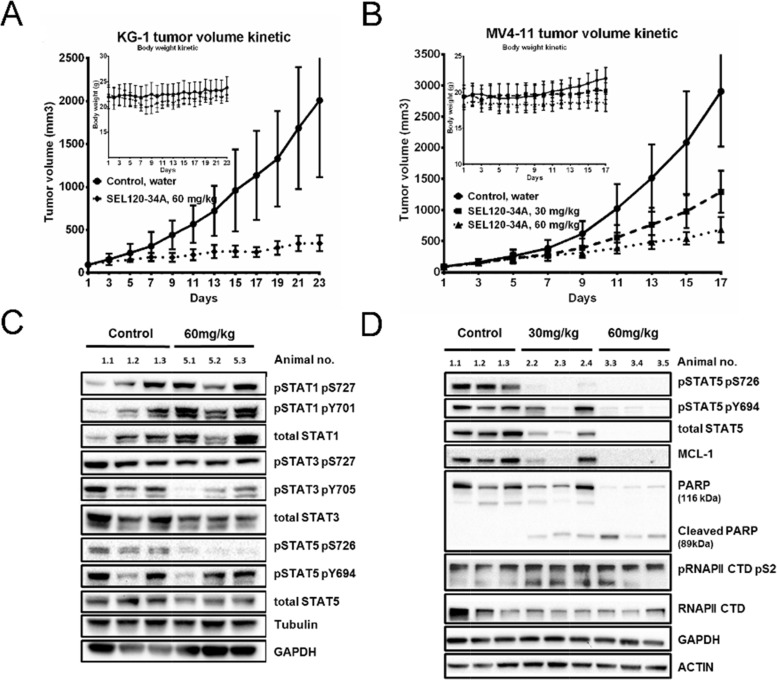
SEL120-34A inhibits growth of AML tumors in a dose- dependent manner **(A)** Volume kinetic of KG-1 tumors and **(B)** MV4-11 tumors grown subcutaneously in SCID mice, treated orally with vehicle (water) or indicated doses of SEL120-34A once every day. Changes in animals’ body weights are shown in the inset; n=8, SD is shown. **(C)** Levels of total STAT1, STAT3 and STAT5 and phosphorylated STAT1 S726, Y701, STAT3 S727, Y705, STAT5 726, Y694 were analyzed in KG-1 tumors from control (vehicle) and SEL120-34A treated animals at day 23. **(D)** Levels of total on STAT5 and phosphorylated STAT5 S726, Y694, MCL-1, PARP and Cleaved PARP, total RNAPII and phosphorylated RNAPII CTD S2 were analyzed in MV4-11 tumors from control (vehicle) and SEL120-34A treated animals at day 17.

Next, we assessed *in vivo* activity of SEL120-34A in xenografted KG-1 and MV4-11 AML tumors grown in SCID mice models. SEL120-34A treatment resulted in completely arrested growth of KG-1-derived tumors and a reduced volume of MV4-11 tumors, without appreciable loss in body weight (Figure 9A and 9B).

High levels of STAT5 S726 in analyzed KG-1 and MV4-11 tumor tissues allowed for pharmacodynamic measurement of target engagement, whereas MOLM13 cells were negative for STAT5 S726 also *in vivo* (Supplementary Figure 5B). We observed dose-dependent inhibition of STAT5 S726 after oral administration of the compound once a day in KG-1, whereas STAT1 S726 was unaffected (Figure 8C). Analysis of MV4-11 indicated that in addition to STAT5 S726, total STAT5 levels were also repressed (Figure 8D). More detailed analysis of MV4-11 tumors indicted repression of oncogenic MCL-1 without any appreciable reduction in phosphorylation levels of RNAPII CTD S2 and clear signs of apoptosis shown by cleaved PARP. Finally, subchronic 14 days SEL120-34A treatment was not associated with significant alterations in the peripheral blood of healthy immunocompetent CD-1 mice (Supplementary Table 8).

## DISCUSSION

SEL120-34A is a novel CDK8 inhibitor with high potency, selectivity and favorable pharmacokinetics and pharmacodynamics. The crystal structure of CDK8/CycC with SEL120-34 gives insight into the binding mode of SEL120-34 and reveals key interaction sites in the protein. It occupies the ATP- binding site and also forms contacts with the front pocket including Arg 356 which is a unique structural feature in the CDK family. Comparing SEL120-34A binding mode with other type I CDK8 inhibitors, reveals that it is the only inhibitor with a high inhibitory potency in the absence of extended decoration protruding from the pocket. The favourable affinity of SEL120-34A to CDK8 can be explained by the high shape complementarity of the molecule with the pocket and several types of formed interactions, including three halogen bonds with the hinge and an ionic interaction with Asp 173.

Functional studies indicated that CDK8 could act as an oncogene in CRC through induction of WNT/β-Catenin signaling [7]. SEL120-34A treatment, in contrast to some other CDK8 inhibitors [14, 21] shows high specificity on STATs genes *in vivo*, without significant interference with other pathways such as WNT/β-Catenin signaling. At the time of writing this manuscript, Clarke et al. published results including transcriptional characterization of Colo-205 tumors treated with two chemically related CDK8 inhibitors [35]. It has been surprising to observe largely distinct effects of these compounds *in vivo* when compared with SEL120-34A. Despite robust inhibition of STAT1 S727 by these compounds, only minor effects on STAT-dependent genes could be observed. In contrast many of the genes which were significantly affected could contribute to the reported toxicity profile.

Specific effects of SEL120-34A on the STAT pathway could be anticipated based on the robust inhibition of phosphorylated STATs. It was astonishing to observe that transcriptional profiling demonstrated such a dominant effect on the IFN-responsive genes *in vivo*, which are generally considered to be a tumor-suppressive pathway in solid tumors [45]. More recently, this simplified view on the role of STAT1 has been challenged by reports indicating that STAT1 up-regulation causes activation of genes which are associated with increased resistance of tumor cells to genotoxic stress and tumor growth in breast cancer [46, 47]. Modulation of response to genotoxic stress has been postulated as one of the therapeutically relevant mechanisms of action for CDK8 inhibitors, which may increase efficacy of cancer chemotherapy [10]. High expression of STAT-dependent genes, many of which are regulated by SEL120-34A *in vivo*, is one of the features of the consensus molecular subtype CMS1 for colorectal cancer, characterized by substantial infiltration of immune cells and a DNA-damage response [48]. The exact role of CDK8 in the process of immune-evasion of cancer cells is still unknown, however it may be partially related to the STAT1 S727 dependent repression of NK-cells cytotoxicity [49]. SEL120-34A, does not affect normal hematopoiesis and could thus be considered for further immunomodulatory studies.

Interestingly, GSEA for SEL120-34A-related transcriptional changes *in vivo*, showed high enrichment of genes involved in the leukemic transformation of human hematopoietic cells by NUP98-HOXA9 chimeric protein resulting from t(7;11)(p15;p15) chromosomal translocation. This genetic rearrangement is the prototype of several NUP98 fusions that occur in AML, where STAT5 rather than STAT1 transcription appears to play a dominant role. Constitutive activation of STAT5 has been observed in the vast majority of AML cases, and repression of STAT5 impairs long-term maintenance of leukemic stem/progenitor cells [50–53]. Recently, pharmacological inhibition of CDK8 has been shown to suppress proliferation in a subset of AML cell lines [4]. Our results recapitulate some of these findings and validate SEL120-34A as a strong inhibitor of proliferation in AML cell lines, both *in vitro* and in *vivo*. The complex nature of these effects is evident given the significant differences in cell lines sensitivity between CA and the three other type I CDK8 inhibitors used in this study. It is intriguing that SEL120-34A and two other inhibitors lack activity on cells previously characterized by the restrained activation of SE-associated genes (such as MOLM-14).

Our results provide, for the first time, evidence for phosphorylated STAT5 and STAT1 as markers for selecting AML cell lines sensitive to treatment with CDK8 inhibitors. At the same time we propose STAT5 S727 as a major pharmacodynamical marker in AML. Activity of STAT5 is often associated with leukemia stem cells phenotype and therefore it has not been surprising in this context to observe that several sensitive AML cells were CD34 positive. Transcriptional profiling of these cells identified gene expression programs associated with stem cells phenotype, whereas resistant cells expressed genes indicating lineage commitment.

Further work is clearly needed in order to establish whether predictive biomarkers for identifying clear responders to CDK8 inhibitors could be applicable in the clinical context.

## MATERIALS AND METHODS

### Chemicals and stock solutions

Reference compounds were synthesized accordingly to procedures described in the patent: Senexin B (Senex Biotechnology Inc.) WO2014/134169A1, whilst synthesis of CCT241545 has already been described [14].

### CDK8 and CDK19 kinase assay

A radiometric protein kinase assay (PanQinase® Activity Assay, ProQinase) was used for measuring activities of CDK8/CycC and CDK19/CycC protein kinases. The CDK8 Eu kinase binding assay (Life Technologies) for CDK8/CycC was performed according to the manufacturer's instructions.

### Kinase capture experiments using ATP-desthiobiothin

Kinase capture experiments were performed using the Kinase Enrichment Kit (Pierce) according to manufacturer's instructions, Briefly, kinases were captured for 10 min with 10 μM ATP desthiobiothin probe in lysis buffer/ 4 M urea. Captured kinases were pulled down with agarose and analyzed by Western blot.

### X-Ray crystallography

Protein expression and purification of the corresponding CDK8 (Hs1-403)/ CycC (Hs1-283) constructs were performed according to previously published protocols [54]. Protein crystals were obtained within the hanging drop setup as described in the literature [54]. Data set collection and processing is described in the supplementary materials and methods.

### Cell cultures

The full list of cell lines used and growth conditions are provided in the supplementary methods. Cell line genotypes were confirmed by multiplex short tandem repeat amplification (GenMed, Poznań, Poland). In some experiments cells were treated with human type I interferon α/β at 250 IU/ml (IFNa) (Sigma) or type II interferon γ (IFNg) at 2.5 ng/ml (BioLegend), as indicated in figure legends.

### Serum, IFNa and IFNg treatment for gene expression measurements

HCT-116 and Colo-205 cells were seeded onto 6-well plates at 2.5×10^5^/well. On the following day, cells were synchronized for 24 h by 0.5% FBS starvation, pretreated with 0.1% DMSO or SEL120-34A at the indicated concentration for 1 h and then supplemented with either 10% FBS, IFNg or IFNa in the presence of the inhibitor. Cells were centrifuged at 1300 rpm for 5 min at 4°C, washed once with 1 ml of ice-cold PBS and stored at -80°C.

### Extended cytotoxicity tests

Cells grown in suspension were plated onto 96-well culture dishes in triplicate at 20000 cells per well in 200 μl of their culture medium, except Kasumi-1 cells, which were plated at 60 000/well. Cells were incubated in the presence of vehicle, 1 μM docetaxel (positive control, Santa Cruz Biotechnology) or 6 serially - diluted concentrations of tested compound. The compound effect was determined by AlamarBlue measurement. On days 3, 6-7 and 10, an equal volume from all wells was also split-back with fresh media supplemented with tested compound, resulting in a cell density for the vehicle well that matched the initial seeding density. For days 6-7, 9-10 and 13-14, the theoretical cell number represents the split-adjusted cell number plotted versus concentration and time. GI_50_ and IC_50_ values were interpolated from dose-response curves for each timepoint fitted by GraphPad Prism 6.

### Xenograft experiments

All animals were handled in strict accordance with good animal practice as defined by the relevant national and local animal welfare bodies. Animals, SCID/beige C.B17 female mice were provided by Harlan Laboratories or Charles River Laboratories, and were maintained in pathogen free conditions.

Mice were inoculated subcutaneously (sc) above the groin on the right hind limb with 4×10^6^ MV-4-11 or KG-1 leukemia cells, 5×10^6^ Colo-205 CRC cells in a 0.1 ml mixture (3:1, v:v) of PBS and Matrigel (BD Biosciences, Growth Factor Reduced, Phenol Red-free; #356231). Once tumors reached a mean volume of approximately 100 mm^3^ for tumor growth assessment or 250mm^3^ for pharmacodynamics assessment, the mice were then randomly divided into uniform groups followed by compound or vehicle administration. Prior to use, the test compound was dissolved freshly in water at indicated doses and administered *per os* (PO) using cannula in a volume of 10 μl per 1 g of body weight. After the last administration, mice were subjected to anesthesia for blood collection, and sacrificed for tumor collection.

### Toxicology assessment

To evaluate toxicity of the SEL120-34A compound, a 14-days toxicology study was run in CD-1 female mice. The day before the first administration of vehicle (BID, PO) or test compound, mice were weighed and randomized into uniform groups (5 mice per group). Prior to use, the test compound was dissolved freshly in water and administered PO at indicated doses in a volume of 10 μl per 1 g of body weight. Body weight and the general condition of the animals were monitored daily. When the study finished on Day 15, mice were subjected to anesthesia, and blood was collected into tubes containing K_2_EDTA for analysis of hematology parameters (ABC Vet Animal Blood Counter).

### Microarray analysis and GSEA

Colo-205 tumours were used for the whole transcriptome analysis. The average signal from the HumanHT-12 v4 Expression BeadChips (AROS Applied Biotechnology, Aarhus N, Denmark) was quantile normalized with no background correction. All computations were performed as previously described [55]. Differentially expressed probe sets were identified using a t-test (Welch variant), followed by the Benjamini-Hochberg *p*-value correction for multiple hypotheses testing. Adjusted *p*-values (*p_adj_*) ≤ 0.05 were considered significant. The log_2_ fold change values of SEL120-34A-treated samples versus control samples were used for the ranking of genes and as an input for the Gene Set Enrichment Analysis (GSEA). The GSEA version 2.2.2 [30] was carried out using the GSEA preranked module. Signatures included curated gene sets (C2 v.5.1) downloaded from MSigDB as well as published data sets [4]. Mapping to Gene Ontology (GO) [56] terms was performed with the GO.db (2.6.1) package [57]. Microarray data were deposited in the Gene Expression Omnibus under GSE77987.

## SUPPLEMENTARY MATERIALS FIGURES AND TABLES





## Abbreviations

GSEA: Gene Set Enrichment Analysis
DEDS: Differential Expression via Distance Summary
NES: Normalized Enrichment Score
AML: Acute Myeloid Leukemia
CRC: Colorectal Cancer
CA: Cortistatin A
F: Flavopiridol
IFNa: human type I interferon α/β
IFNg: type II interferon γ
IER: Immediate Early Response
PO: per os
SC: subcutaneous
CCLE: Cancer Cell Line Encyclopedia
