# Supplementary material for: SEL120-34A is a novel CDK8 inhibitor active in AML cells with high levels of serine phosphorylation of STAT1 and STAT5 transactivation domains
Source: Oncotarget. 2017 Apr 4;8(20):33779–95. doi: 10.18632/oncotarget.16810 (PMC5464911; doi:10.18632/oncotarget.16810)
Supplement: Supplementary file 1 [file oncotarget-08-33779-s001.pdf]

SEL120-34A is a novel CDK8 inhibitor active in AML cells with high levels of serine phosphorylation of STAT1 and STAT5 transactivation domains

SUPPLEMENTARY DATA

X-Ray crystallography

Data sets were collected at beamline S06SA of the Swiss Light Source using cryogenic conditions. Data were processed using XDS and XSCALE [1]. Molecular replacement was performed by MOLREP [2]. Iterative model building was carried out with Coot [3], coupled with refinements using Refmac5 [4]. The best structure of CDK8/CycC complexed with SEL120-34A was resolved at 2.8 Å resolution (details on crystallization, structure determination and refinement are in the (Supplementary Table 1). For calculating the free R-factor, a measure to cross-validate the validity of the final model, about 3.6 % of measured reflections were excluded from the refinement procedure (Supplementary Table 2).

Cell cultures

The following cell lines were used: Colo-205 (ATCC; 90% RPMI/10% FBS), EoL-1 (Sigma Aldrich; 90% RPMI/10% FBS), GDM-1 (ATCC; 90% RPMI/10% FBS), HCT-116 (DSMZ; 90% DMEM high glucose/10% FBS), HEL92.1.7 (DSMZ; 90% RPMI/10% FBS), HL-60 (ATCC; 90% RPMI/10% FBS), Kasumi-1 (DSMZ; 80% RPMI/20% FBS), KG-1 (DSMZ; 90% RPMI/10% FBS), KU-812 (DSMZ; 80% RPMI/20% FBS), MOLM-6 (DSMZ; 80% RPMI/20% FBS), MOLM-13 (DSMZ; 90% RPMI/10% FBS), MOLM-14 (DSMZ; 90% RPMI/10% FBS), MOLM-16 (DSMZ; 80% RPMI/20% FBS), MV-4-11 (CLS; 90% IMDM/10% FBS), OciAML-2 (DSMZ; 90% α-MEM/10% FBS), OciAML-3 (DSMZ; 80% α-MEM/20% FBS), OciAML-5 (DSMZ; 80% α-MEM/20% FBS), SET-2 (CLS; 90% RPMI/10% FBS), SKNO-1 (DSMZ; 90% RPMI/10% FBS + 10 ng/ml GM-CSF), THP-1 (CLS, 90% RPMI/10% FBS), ME-1 (DSMZ; 90% RPMI/10% FBS + 10 ng/ml GM-CSF), PL21 (DSMZ; 80% RPMI/20% FBS), SIG-M5 (DSMZ; 80% RPMI/20% FBS), SKM-1 (DSMZ; 80% RPMI/20% FBS).

To measure protein levels, cells were seeded onto 6-well plates at $2x10^6$/well (cultures grown in suspension) or $0.5x10^6$/well (adherent cultures), treated with compounds as indicated in figure legends and harvested in RIPA buffer. Adherent cell lines were allowed to attach to the surface of culture dishes before treatments.

Quantitative reverse transcriptase PCR (qRT-PCR)

One microgram of total RNA and random hexamers were used to synthesize cDNA with Superscript III (Life Technologies), according to the manufacturer's protocol. qPCR on the cDNA was performed using an Applied Biosystems 7900HT Fast Real-Time PCR System and a Sensimix SYBR Kit (Bioline). RPLP0 was used as the reference mRNA. Gene expression results were calculated using the ΔΔCt method [5]. Differences were evaluated using the Mann–Whitney U-test by GraphPad Prism 5 (GraphPad Software). A p-value of less than 0.05 was considered statistically significant.

SDS-PAGE, immunoblotting and antibodies

Cells were harvested at indicated timepoints in RIPA buffer (50 mM Tris-HCl pH 8.0; 150 mM NaCl; 5 mM EDTA; 1% NP.-40; 0,5% sodium deoxycholate;

List of primers used in qPCR

Gene	Assay	Localization	Forward (5'-->3')	Reverse (5'-->3')
**EGR1**	RT, mRNA	ex1-ex2	CAGCACCTTCAACCCTCAG	AGCGGCCAGTATAGGTGATG
**FOS**	RT, mRNA	ex1-ex2	GTGGGAATGAAGTTGGCACT	CTACCACTCACCCGCAGACT
**RPLP0**	RT, mRNA	ex7-ex8	GCAATGTTGCCAGTGTCTG	GCCTTGACCTTTTCAGCAA
**STAT1**	RT, mRNA	ex4-5	TGAATATTCCCCGACTGAGC	AGGAAGACCCAATCCAGATGT
**IRF9**	RT, mRNA	ex1-2	GCCCTACAAGGTGTATCAGTTG	TGCTGTCGCTTTGATGGTACT
**ISG15**	RT, mRNA	ex1-2	ACTCATCTTTGCCAGTACAGGAG	CAGCATCTTCACCGTCAGGTC

0,1% SDS; 20 mM β-glicerol phosphate; 5 mM NaF; 1 mM Na_3VO_4) supplemented with protease inhibitors (Santa Cruz Biotechnology). Tumor samples were homogenized in 15 sec intervals and kept on ice between homogenization cycles. Protein concentration in lysates was measured by the Bradford assay and adjusted to achieve the same final concentration in each well (20-30 µg protein/well). Lysates were resolved on 10% Mini-PROTEAN TGX precast gels (Biorad) and transferred using the Trans-Blot Turbo Transfer System (Biorad). Unless indicated otherwise, membranes were blocked for 45 min with 2% fat-free dried milk (Rovema, Poland) in Tris-buffered saline (TBS) + 0.05% Tween (TBST), incubated overnight at 4°C with primary antibodies diluted into 2% milk in TBST and washed four times for 10 min with TBST. This was followed by a 1h room-temperature incubation with secondary donkey anti-rabbit antibodies conjugated to HRP, used at 1:4000 (Cell Signaling, Beverly, MA; cat # 7074), 1:8000 (Millipore; cat # AP18P) or 1:20000 (Sigma Aldrich; cat. # 0545). Membranes were washed four times for 10 min in TBST and developed with an ECL reagent (GE Healthcare, Buckinghamshire, UK) on X-ray film (GE Healthcare) or in the ChemiDoc MP Imaging System (BioRad).

Primary antibodies used for immunoblotting were: α-CDK8 (1:1000; Cell Signaling, cat. # 4106), α-CDK9 (1:1000; Cell Signaling, cat. # 2316), α-CDK19 (1:500; Sigma Aldrich, cat. # HPA007053), α-ERK ½-pT202/T204 (1:1000, Cell Signaling cat. # 4377), α-ERK ½ (1:1000, Cell Signaling cat. # 4695), α-Pol II CTD-pS2 (1:1000; Abcam, cat. # ab5095), α-Pol II CTD-pS5 (1:1000; Abcam, cat. # ab5131), α-STAT1 (1:1000; SAB Biotech, cat. # 21044), α-STAT1-pS727 (1:500; Cell Signaling, cat. # 9177), α-STAT1-pY701 (1:1000; SAB Biotech, cat. # 11044), α-STAT5 (1:1000; Santa Cruz, cat. # sc835), α-STAT5-pS726 (1:1000; Abcam, cat. # ab128896), α-STAT5-pY694 (1:1000; Cell Signaling, cat. #C11C5), α-tubulin (1:4000; Sigma, cat. # T5192) and β-actin (1:4000, Sigma cat. # A2066) or GAPDH (1:20000; Sigma, cat. # G9545)

mRNA extraction from xenograft tissue

Following extraction, tumors were preserved in RNAlater (Qiagen) and stored at -80°C. A scoop of a tumor (~25mg) was homogenized with a pestle under liquid nitrogen and immediately processed according to the RNeasy Mini Kit protocol (Qiagen). The RNA concentration was measured by a NanoDrop spectrophotometer and stored at -80°C for downstream analyses. Prior to microarray analyses, the quality of the RNA samples was determined using an Agilent 2100 Bioanalyzer (Agilent Technologies, CA, USA).

Chemical synthesis of SEL120-34A

SEL120-034A **1** could be easily assembled by 5-step synthesis starting from commercially available 7-methyl-8-nitroqunioline **(2)** as shown in Scheme 1. After reduction of 7-methyl-8-nitroqunioline **(2)** with Adams's catalyst obtained 7-methyl-1,2,3,4-tetrahydroquinolin-8-amine **(3)** was used immediately for the cyclisation reaction. The heating of **3** with urea gives quinolinone derivative **4** which was brominated with NBS. The reaction of **6** with Boc-piperazine followed by deprotection provides to the final compound **1**. The preparation of **1** was performed in an efficient sequence with an overall yield 40% starting from easily accessible building block. Moreover developed synthetic pathway allowed for a multigram scale synthesis of compound **1**.

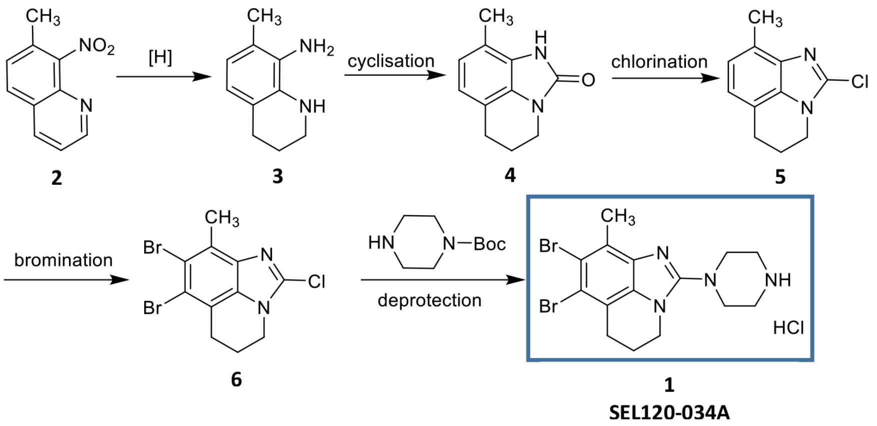

Scheme 1: Total synthesis of SEL120-34A.

REFERENCES

1. Kabsch W. XDS. Acta Crystallogr D Biol Crystallogr. England; 2010; 66: 125–32. doi: 10.1107/S0907444909047337.
2. Vagin A, Teplyakov A. Molecular replacement with MOLREP. Acta Crystallogr D Biol Crystallogr. England; 2010; 66: 22–5. doi: 10.1107/S0907444909042589.
3. Emsley P, Cowtan K. Coot: model-building tools for molecular graphics. Acta Crystallogr D Biol Crystallogr. Denmark; 2004; 60: 2126–32. doi: 10.1107/S0907444904019158.
4. Vagin AA, Steiner RA, Lebedev AA, Potterton L, McNicholas S, Long F, Murshudov GN. REFMAC5 dictionary: organization of prior chemical knowledge and guidelines for its use. Acta Crystallogr D Biol Crystallogr. Denmark; 2004; 60: 2184–95. doi: 10.1107/S0907444904023510.
5. Livak KJ, Schmittgen TD. Analysis of relative gene expression data using real-time quantitative PCR and the 2(-Delta Delta C(T)) Method. Methods San Diego Calif. 2001; 25: 402–8. doi: 10.1006/meth.2001.1262.

SUPPLEMENTARY FIGURES AND TABLES

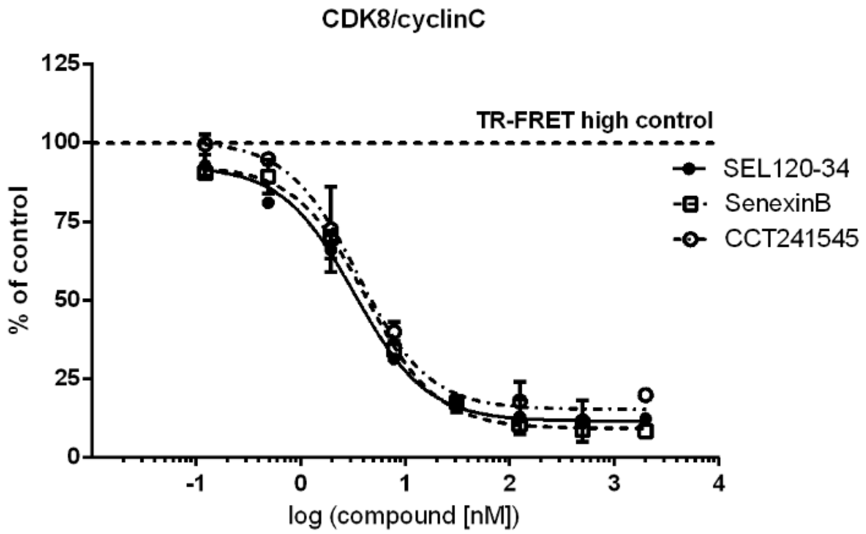

	SEL120-34	SenexinB	CCT241545
HillSlope	-1.2	-1.3	-1.3
IC50	3.3	4.2	3.6

Supplementary Figure 1: SEL120-34A binding to CDK8/CycC. The IC_{50} of SEL120-34A, Senexin B and CCT241545 determined by constructing a dose-response curve and examining binding to CDK8/CycC in the LanthaScreen TR-FRET assay (LifeTechnologies)

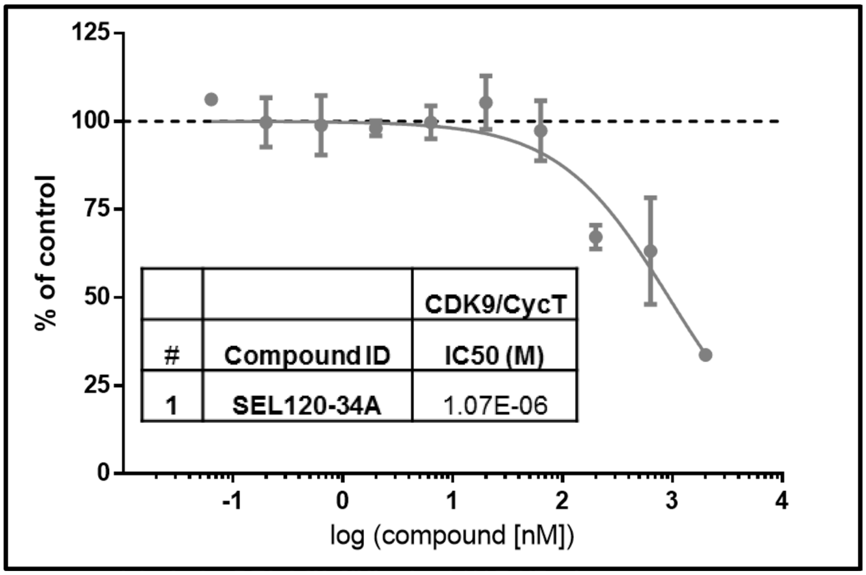

Supplementary Figure 2: Activity of CDK9/CycT1 in the presence of SEL120-34A. The IC_{50} of SEL120-34A determined by constructing a dose-response curve and examining inhibition of CDK9/CycT1 at Km ATP concentration (ProQinase).

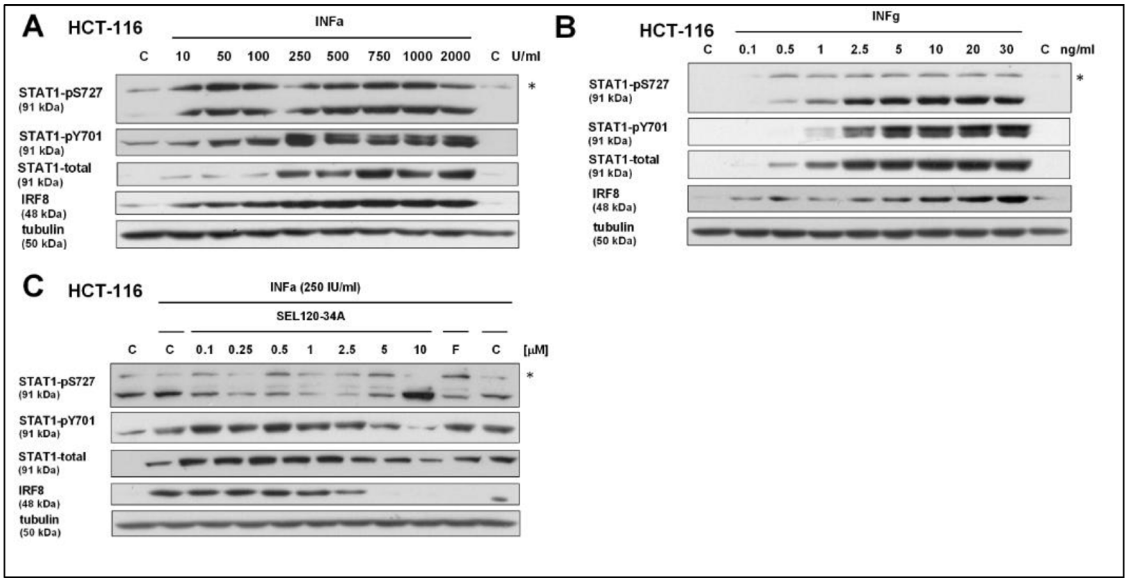

Supplementary Figure 3: Induction of JAK/STAT signaling in CRC cells treated with interferons. HCT-116 cells were treated with increasing amounts of IFNa **(A)** and IFNg **(B)** protein levels of STAT1 pS727, pY701, total STAT1, IRF8 and tubulin were measured by WB. **(C)** The SEL120-34A compound inhibits interferon-dependent induction of STAT1 pS727. HCT-116 cells stimulated with INFa for 4 h were treated with increasing doses of SEL120-34A or Flavopiridol (F) to determine changes in phosphorylation of STAT1 pS727, STAT1 pY701 and IRF8, as measured by WB.

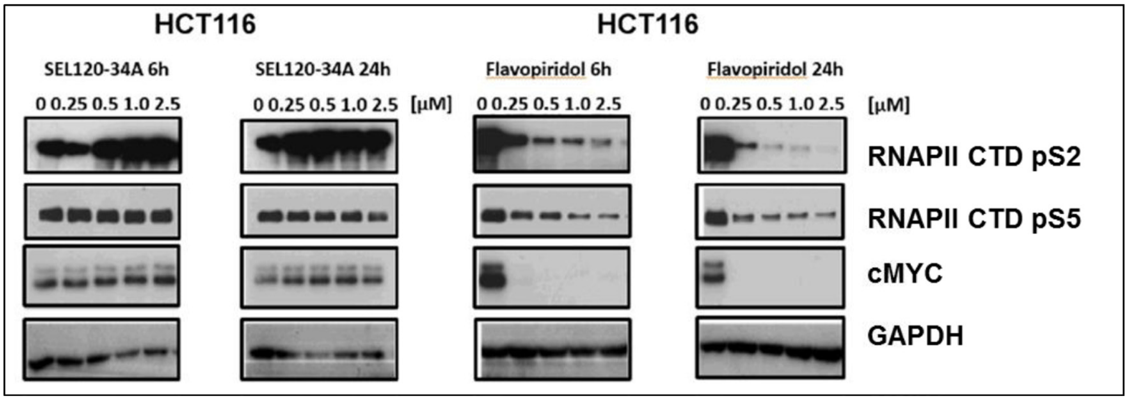

Supplementary Figure 4: Treatment with SEL120-34A does not inhibit phosphorylation of RNAP II CTD associated with the activities of CDK9 in cells. HCT-116 cells were treated with indicated concentrations of SEL120-34A and Flavopiridol for 6 h and 48 h. Protein levels of RNAP II CTD pS2, pS5, cMYC and GAPDH were measured by WB.

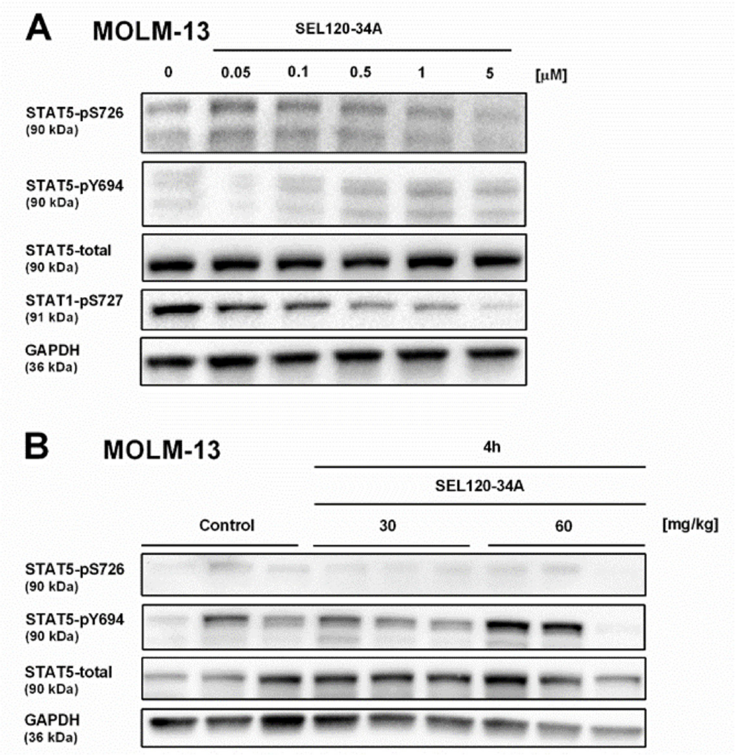

Supplementary Figure 5: STAT5 signaling is inactive in MOLM-13, a non-responder AML cell line. (A) Protein levels of STAT5 S726, Y694, total STAT5, STAT1 S727 and GAPDH in MOLM-13 cells treated with SEL120-34A for 24 h and measured by WB **(B)** Protein levels of STAT5 S726, Y694, total STAT5 levels in xenografted MOLM-13 cells treated with vehicle for three days BID with 30 and 60 mg/kg doses of SEL120-34A and measured by WB.

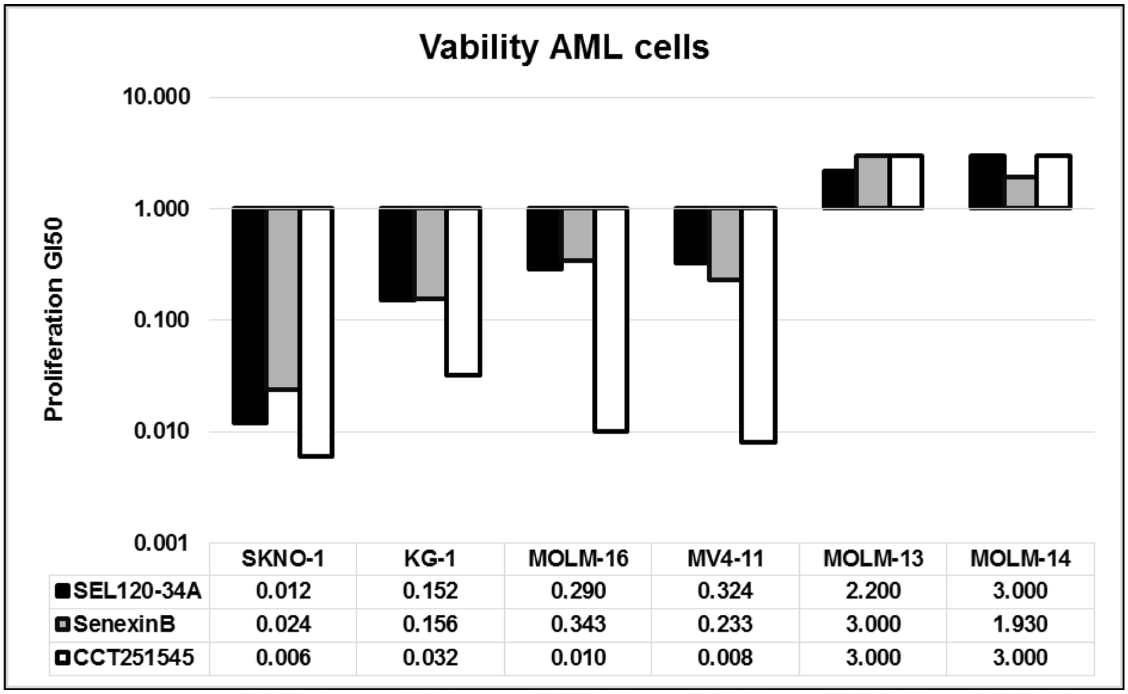

	SKNO-1	KG-1	MOLM-16	MV4-11	MOLM-13	MOLM-14
SEL120-34A	0.012	0.152	0.290	0.324	2.200	3.000
SenexinB	0.024	0.156	0.343	0.233	3.000	1.930
CCT251545	0.006	0.032	0.010	0.008	3.000	3.000

Supplementary Figure 6: Sensitivity pattern of three structurally non-related CDK8 inhibitors is similar in AML cells. Effects of SEL120-34A, SenexinB and CCT251545 on growth of indicated AML cell lines, measured by extended viability tests. GI_{50} values [µM] (day 10) are shown and plotted on the graph.

Supplementary Table 1: CDK8 crystallization: Data collection and processing

X-ray source	PXI/X06SA (SLS)*
Wavelength [Å]	0.99988
Detector	PILATUS 6M
Temperature, [K]	100
Space group	$P\ 2_1\ 2_1\ 2_1$
Cell: a, b, c, [Å]	70.78, 71.16, 170.97
α, β, γ, [°]	90.0, 90.0, 90.0
Resolution, [Å]	2.81 (3.06-2.81)
Unique reflections	21,007 (4702)
Multiplicity	2.8 (2.9)
Completeness, [%]	96.6 (97.7)
R_{sym}, [%]	7.6 (44.1)
R_{meas}, [%]	9.4 (53.8)
Mean(I)/SD	13.10 (3.02)

* Swiss Light Source (SLS, Villigen, Switzerland) Values in parentheses refer to the highest resolution bin Rmeas is calculated from independent reflections.

Supplementary Table 2: Refinement statistics

**Resolution, [Å]**	**85.55-2.81**
Number of reflections (working/test)	20,253 / 753
R_{cryst}, [%]	21.1
R_{free}, [%]	27.8
Total no. of atoms:	
Protein	5025
Water	74
Ligand	21
Deviation from ideal geometry	
Bond length, [Å]	0.007
Bond angles, [°]	1.05
Bonded B's, [Å]	5.3
Ramachandran plot:	
Most favoured regions, [%]	92.8
Additional allowed regions, [%]	6.4
Generously allowed regions, [%]	0.4
Disallowed regions, [%]	0.4

The final model, which comprises residues for CDK8 and CycC according to sequences P49336 and P24863 (Uniprot) and its completeness as defined by electron density are as follows : chain A (CDK8 residues 1-360) except for the segments 115-120, 186-195, 239-243, chain B (CycC residues -1-264). Additional residues were introduced (termed -1, 0 for CycC where 1 refers to $Met1^{CycC}$) at the N terminus during cloning at the cleavage site.

Supplementary Table 3: List of probesets differentiating between control and SEL120-34A treated Colo205 xenografts

See Supplementary File 1

Supplementary Table 4: Gene ontology (GO) terms corresponding to the top 10% percent of probe sets that differentiated between control and SEL120-34A treated Colo205 xenografts. Probe sets were sorted according to their p-value and were used as input for GO analyses

See Supplementary File 1

Supplementary Table 5: Gene Set Enriched Analysis (GSEA) Colo205 tumors treated with SEL120-34A 30mg/kg - curated gene sets (C2 v.5.1)

See Supplementary File 1

Supplementary Table 6: Gene Set Enriched Analysis (GSEA) Responder cells- curated gene sets (C2 v.5.1)

See Supplementary File 1

Supplementary Table 7: Gene Set Enriched Analysis (GSEA) Non-responder cells- curated gene sets (C2 v.5.1)

See Supplementary File 1

Supplementary Table 8: Blood morphology of CD1 mice treated with SEL120-34A. Total blood cell counts were assessed in CD-1 mice treated with SEL120-34A for 14 days. No statistical differences were shown between control group and administrated groups (t-test) in respective parameters. RBC – red blood cells, HGB – hemoglobin, HCT – hematocrit, WBC – white blood cells, LYMPH – lymphocytes, MONO – monocytes, GRA – granulocytes, PLT – platelet, MPV – mean platelet volume

See Supplementary File 1
